# COVID-19 Infection: Its Lingering Symptoms in Adults

**DOI:** 10.7759/cureus.24736

**Published:** 2022-05-04

**Authors:** Durgamani Kishore Yellumahanthi, Bethany Barnett, Sarabeth Barnett, Shushruth Yellumahanthi

**Affiliations:** 1 Family Medicine, University of Alabama at Birmingham, Huntsville, USA; 2 Family Medicine, Family Medicine of Huntsville, Huntsville, USA; 3 None, Ardmore High School, Ardmore, USA; 4 None, New Century Technology High School, Huntsville, USA

**Keywords:** lingering symptoms covid, long-covid-19, long-haul covid, long term side effects covid, long covid

## Abstract

Background

Recent studies showed that a significant percentage of people who recovered from coronavirus disease 2019 (COVID-19) had lingering symptoms. Among patients diagnosed with COVID-19 infection, studies showed persistent symptoms both in patients hospitalized and in outpatient settings. In the studies done in the outpatient setting involving mild to moderate COVID-19 patients, there were significant variations regarding the exact percentage of people with lingering symptoms. Also, in the outpatient setting, not many studies were done on COVID-19 patients that assessed risk factors for having lingering symptoms. Given that a large percentage of people infected with COVID-19 infection do not get hospitalized, it is imperative that this lacuna be filled. We believe knowing the details of long-term symptoms of COVID-19 infection both from prevalence and predictors point of view, could allow the physicians, healthcare system and community to better prepare for managing and following these patients.

Materials and methods

Our study period was within 12 months after the first documented case of COVID-19 occurred in the State of Alabama. Our study population included patients who were diagnosed with a documented case of COVID-19 in this time period and were under the care of a single primary care provider at an ambulatory clinic. Among 80 patients who had documented COVID-19, three left the practice, two declined to participate in the study and three were deceased (two due to COVID-19 and one for other reasons). Therefore, the study population constituted 72 patients. A questionnaire was mailed to all 72 patients to see how many of them had symptoms three months and beyond of having COVID-19 infection. A chart review was conducted for the study participants to assess for “Comorbid conditions”, health conditions that were considered conclusively high risk for acute COVID-19 infection by US Center for Disease Control and Prevention (CDC).

Results

Fifty-three patients responded to the questionnaire; 27 patients (50.9%) reported lingering symptoms beyond three months of diagnosis with COVID-19 infection. The three most common symptoms reported were fatigue (56%), brain fog (48%), and shortness of breath (41%). The results also showed that women are more likely than men to have lingering symptoms. “Elderly” (≥65 years) patients were as likely as 18-64 years old patients to have lingering symptoms and the presence of one or more of the “Comorbid conditions” does not have any bearing on the occurrence of lingering symptoms.

Conclusion

Future studies should be done in a larger population to assess the findings that our study showed regarding “elderly” age and the presence of one or more “comorbid conditions” being independent variables of the occurrence of prolonged COVID-19 symptoms.

We recommend studies be done assessing the prevalence and predictors for the long-term effects of the COVID-19 infection. This knowledge could help in preventing those long-term symptoms from occurring in the first place and also in preparing the patient, the physician and the community in managing the outcomes effectively.

## Introduction

Recent studies showed that a significant percentage of people who recovered from coronavirus disease 2019 (COVID-19) had lingering symptoms several weeks to months after severe acute respiratory syndrome coronavirus 2 (SARS-CoV-2) infection [1-4]. Long COVID-19, COVID-19 long-haulers, post-COVID-19 condition and post-acute sequelae of SARS-CoV-2 infection are some of the terms that were introduced in the literature to describe illness in persons who continue to report lasting effects several weeks to months after SARS-CoV-2 infection [5-9].

Among patients diagnosed with COVID-19, studies showed persistent symptoms both after hospitalization [1,10] and in outpatient settings [9,11-13]. In the outpatient setting involving mild to moderate COVID-19 patients, there were significant variations regarding the exact percentage of people with lingering symptoms [3,4,14]. Also, not many studies were done in the outpatient scenario that assessed risk factors for having lingering symptoms. Given that a large percentage of people infected with COVID-19 infection do not get hospitalized, it is imperative that this lacuna be filled.

We believe that knowing the symptoms of long-term COVID-19 would allow physicians, other healthcare providers, and the community better prepare for the management of long-term COVID-19 patients.

## Materials and methods

Our study was submitted to the Institutional Review Committee (IRC), Huntsville Hospital, Huntsville, Alabama and was approved for exemption from IRC review.

Study period

Within 12 months after the first documented case of COVID-19 occurred in the State of Alabama (March 13, 2020 to March 12, 2021). 

Study population

All documented COVID-19 patients during the study period belonged to a single primary care provider working at an ambulatory clinic in Huntsville, Alabama.

Eighty patients had documented COVID-19 during this study period. Among those, three left the practice, two declined to participate in the study, and three were deceased (two due to COVID-19 and one for other reasons). Therefore, the study population constituted 72 patients. A questionnaire was mailed to all 72 patients to see how many had symptoms three months and beyond of having COVID-19. 

A chart review was conducted for the study participants to assess for “Comorbid conditions”, health conditions that were considered high risk for acute COVID-19 infection by US Center for Disease Control and Prevention (CDC). Given that this list is getting modified constantly based on new evidence, our study considered only those comorbidities that are currently listed under the category “Supported by at least one meta-analysis or systemic review or by review method defined in scientific evidence” (Table 1).

**Table 1:** Conclusive medical conditions that increase a person’s risk of severe illness from COVID-19 per CDC.

Bronchiectasis
Cancer
Cerebrovascular disease
Chronic kidney disease
Chronic liver disease (cirrhosis, non-alcoholic fatty liver disease, alcoholic liver disease, autoimmune hepatitis)
COPD
Cystic fibrosis
Diabetes mellitus, type 1
Diabetes mellitus, type 2
Disabilities, including Down Syndrome
HIV
Heart conditions (such as heart failure, coronary artery disease, or cardiomyopathies)
Interstitial lung disease
Mental health conditions (such as mood disorders, including depression, and schizophrenia spectrum disorders)
Neurologic conditions (Dementia)
Obesity
Physical Inactivity
Pregnancy and Recent Pregnancy
Primary Immunodeficiencies
Pulmonary hypertension and pulmonary embolism
Smoking, current and former
Solid organ or blood stem cell transplantation
Tuberculosis
Use of corticosteroids or other immunosuppressive medications

## Results

Fifty-three patients responded to the questionnaire; 24 were males and 29 were females. Caucasians comprised 51 of the test subjects. African Americans and Asians comprised one each. 38 patients were between 18 and 64 years and 15 were 65 years and above (Elderly). Among the 53 patients, 35 patients had one or more of the “Comorbid conditions.” Four patients were hospitalized. 

Among the 53 patients, 27 patients (50.9%) reported lingering symptoms beyond three months of diagnosis with Covid-19 infection. Fatigue (56%), Brain fog (48%), and Shortness of breath (41%) were the three most common symptoms that were reported. The other significant symptoms that occurred were joint pains (26%), loss of taste or smell (14.8%), anxiety or depression (14.8%), loss of hair (14.8%), sleep disturbances (14.8%) and cough (11.1%).

A chi-square test and Fischer’s exact test were done to see if the manifestation of lingering symptoms can be determined by gender, presence of “Comorbid conditions” and “elderly” age group. The results showed that women are more likely than men to have lingering symptoms. The “Elderly” were as likely as 18-64 years old patients to have lingering symptoms and the presence of one or more “Comorbid conditions” did not have any bearing on the occurrence of lingering symptoms (Table 2).

**Table 2:** The prevalence of lingering symptoms based on gender, age, presence of “Comorbid conditions’’ and their respective Chi square and Fischer’s exact p-values.

Category		Lingering symptoms present	Lingering symptoms absent	p value for Chi square	p value for Fischer’s exact
Gender	Total Males: 24	8	16	0.02	0.028
	Total Females:29	19	10		
Age	Total 18-64 years: 38	20	18	0.696	0.766
	Total >=65 years:15	7	8		
‘Comorbid conditions’	Total Present: 35	18	17	0.926	1.000

## Discussion

Cirulli et al. [15] in their study comprising of a cohort of 357 COVID-19 positive cases, mentioned that 14.8% still had at least one symptom after 90 days. In the cross-sectional study conducted by Lemhofer et al. [14], of the 1,027 patients with mild or moderate COVID-19, 61.9% reported persisting symptoms more than 3 months after infection. Petersen et al. [4] in their study consisting of 180 participants reported that 53.1% had persistence of at least one symptom after a mean of 125 days after symptoms onset. Our study results of 50.9% falls within that range. 

Fatigue, similar to other studies, was the most common lingering symptom in our study [4,14]. Nehme et al. [3] reported that among 669 patients in their study, fatigue, dyspnea, and loss of taste or smell were the main persistent symptoms. A meta-analysis of 15 studies on post-acute sequelae of SARS-CoV-2 showed that among patients aged 17 to 87 years, fatigue, attention disorder, hair loss, and dyspnea were among the commonly reported symptoms [9]. The persistent lingering symptoms consisting of fatigue, brain fog, shortness of breath, loss of taste (or smell), anxiety (or depression), loss of hair, sleep disturbances and cough reported in our study were among the symptoms reported by earlier studies [10,16].

The studies showed that persons with mild to moderate COVID-19 in an outpatient scenario had various long-term symptoms for varying lengths of time [16]. However, no clear understanding exists regarding the predictors of these long-term symptoms and also there seems to be a paucity of the studies conducted to know these predictors. 

We believe it is essential that studies are carried out assessing for predictors for long-term symptoms. This knowledge could help in preventing those long-term symptoms from occurring in the first place. If not, it could at least help in knowing which patients affected with COVID-19 would be a potential candidate for these long-term symptoms and that helps both the patient and the physician in better preparing for those outcomes. This, in turn, could help in more effective management of those long-term symptoms.

Our study assessed gender, elderly age and the presence of comorbid conditions to see if they have any bearing on the occurrence of long-term symptoms among those who recovered from mild to moderate COVID-19 infection in an outpatient setting. Our study results showed that women are more likely than men to have lingering symptoms. In line with our results, Augustin et al. [17] reported that females are more likely to develop lingering symptoms than males.

The general thinking among people is that elderly and those with comorbid conditions are more likely to have lingering problems after recovery from mild to moderate COVID-19 infection. Therefore, we tested that notion. Our study results showed that elderly patients who are ≥65 years are as likely as younger adults (18-64 years) to have lingering symptoms and that the presence of one or more of the comorbid conditions does not have any bearing on the occurrence of lingering symptoms. To our knowledge, no studies were done in an outpatient setting similar to our analysis of “elderly” and “comorbid conditions.” 

We recommend future studies be done with a large power, to assess the findings that our study showed regarding ‘’elderly’’ age and presence of one or more of “comorbid conditions” being independent of the occurrence of lingering symptoms. 

The younger adults (18-64 years) and people with no comorbid conditions are at lesser risk of acquiring COVID-19 infection and also have relatively better prognosis with regards to recovering from acute COVID-19 infection. However, if future studies show that the younger adults and those patients without comorbid conditions are as susceptible as the “elderly” and people with comorbid conditions to have long term symptoms of COVID-19 infection, it could motivate the younger adults and those without comorbid conditions to receive COVID-19 vaccinations and comply with public health measures.

The limitations of our study are small sample size, predominantly constitution of one race (Caucasians) and potential bias from self-reported symptoms.

## Conclusions

We recommend studies be continuously done assessing the prevalence and predictors for the long-term effects of the COVID-19 infection. This knowledge could help in preventing those long-term symptoms from occurring in the first place and also in preparing the patient, the physician and the community in managing the outcomes effectively. In addition, knowing about the long-term symptoms of COVID-19 infection could help in estimating the true burden that it causes on healthcare.
